# The physiology of learning: strategies clinical teachers can adopt to facilitate learning

**DOI:** 10.1007/s00431-021-04054-7

**Published:** 2021-03-29

**Authors:** Satid Thammasitboon, Paul L. P. Brand

**Affiliations:** 1 Intensive Care Medicine Section and Center for Research, Innovation and Scholarship in Medical Education (CRIS), Texas Children’s Hospital, Baylor College of Medicine, Houston, TX USA; 2 Isala Academy, Department of Medical Education and Faculty Development, Isala Hospital, Zwolle, The Netherlands; 3 Lifelong Learning Education and Assessment Research Network (LEARN), University of Groningen and University Medical Center, Groningen, The Netherlands

**Keywords:** Clinical teaching, Adult learning principles, Learning environment, Active learning, Deliberate practice

## Abstract

Almost all pediatricians working in a hospital or office environment have teaching responsibilities to learners such as medical students and residents. Although teaching and supporting learning in a busy work environment imposes challenges to clinical teachers, these clinical settings provide an ideal setup for experiential learning, learning from daily experiences with patients. Advances in the science of learning derived from various fields have informed us how adults learn best. Many techniques and strategies based on this “physiology of learning” have shown their educational values in everyday pediatric practice. This article outlines how clinical teachers can create the conditions to optimize experiential learning for individual or a group of learners. We highlight practical implications of educational theories and evidence-based educational practices for clinical teachers seeking to enhance their teaching effectiveness. These include promoting active learning and engaging learners in deliberate practice; retrieval of knowledge and prior experiences to enhance motivation; supporting a psychologically safe learning environment; helping learners to set goals; fostering collaborative learning; structuring teaching to link it to authentic roles and tasks; and customizing content to individual learners.

*Conclusion*: Applying adult learning principles in everyday teaching activities will support busy pediatricians to be successful in their tasks as clinical teachers, and contribute to work satisfaction.

**Table:** 

**What is Known:** *• Most pediatricians provide clinical teaching to medical students and residents, but few have had formal training in educational techniques.* *• Learning from clinical experiences (experiential learning) is of key importance to becoming and maintaining a competent pediatrician.*	
**What is New:** *• This review presents an up-to-date overview of the physiology of learning, i.e., how people learn.* *• Knowledge of the principles of how people learn helps pediatricians shape their clinical teaching effectively and contribute to their work satisfaction.*

**What is Known:**

*• Most pediatricians provide clinical teaching to medical students and residents, but few have had formal training in educational techniques.*

*• Learning from clinical experiences (experiential learning) is of key importance to becoming and maintaining a competent pediatrician.*

**What is New:**

*• This review presents an up-to-date overview of the physiology of learning, i.e., how people learn.*

*• Knowledge of the principles of how people learn helps pediatricians shape their clinical teaching effectively and contribute to their work satisfaction.*

Scenario:

*Each morning after handover, there is a planned 30-min teaching session to discuss an interesting case from the handover with a group of learners (i.e., three interns, one upper-level resident, a nurse practitioner, and a fellow). This morning, most discussion during handover was about the use of high-flow nasal oxygen (HFNO) in an infant with bronchiolitis. How do you approach this teaching session with this group with different levels of learners?*

## Introduction

Providing teaching and learning in a busy work environment imposes challenges to clinical teachers. At the same time, these busy clinical settings provide an ideal setup to learn from the daily experiences with patients that are encountered by medical students, interns, and residents. This process of learning from relevant experiences is called experiential learning [1, 2]. According to Knowles’ adult learning principles, the participants in experiential learning are self-directed learners who can identify their own learning needs; they use their experience as a basis for their learning; they are oriented to learning things that are immediately applicable; and they prefer material that is relevant to their everyday practice [3]. Over the past decades, advances in the science of learning derived from cognitive psychology, neuroscience, sociology, anthropology, educational science, and behavioral economics have informed us how people learn best. Many techniques and strategies based on this “physiology of learning” have shown their educational values in authentic learning environments such as everyday pediatric practice [4].

We describe here how clinical teachers can create the conditions to optimize experiential learning for individual or a group of learners. We highlight practical implications of educational theories and evidence-based educational practices for clinical teachers seeking to enhance their teaching effectiveness.

## Using the “physiology of learning” in clinical teaching practice

**Fig. 1:**
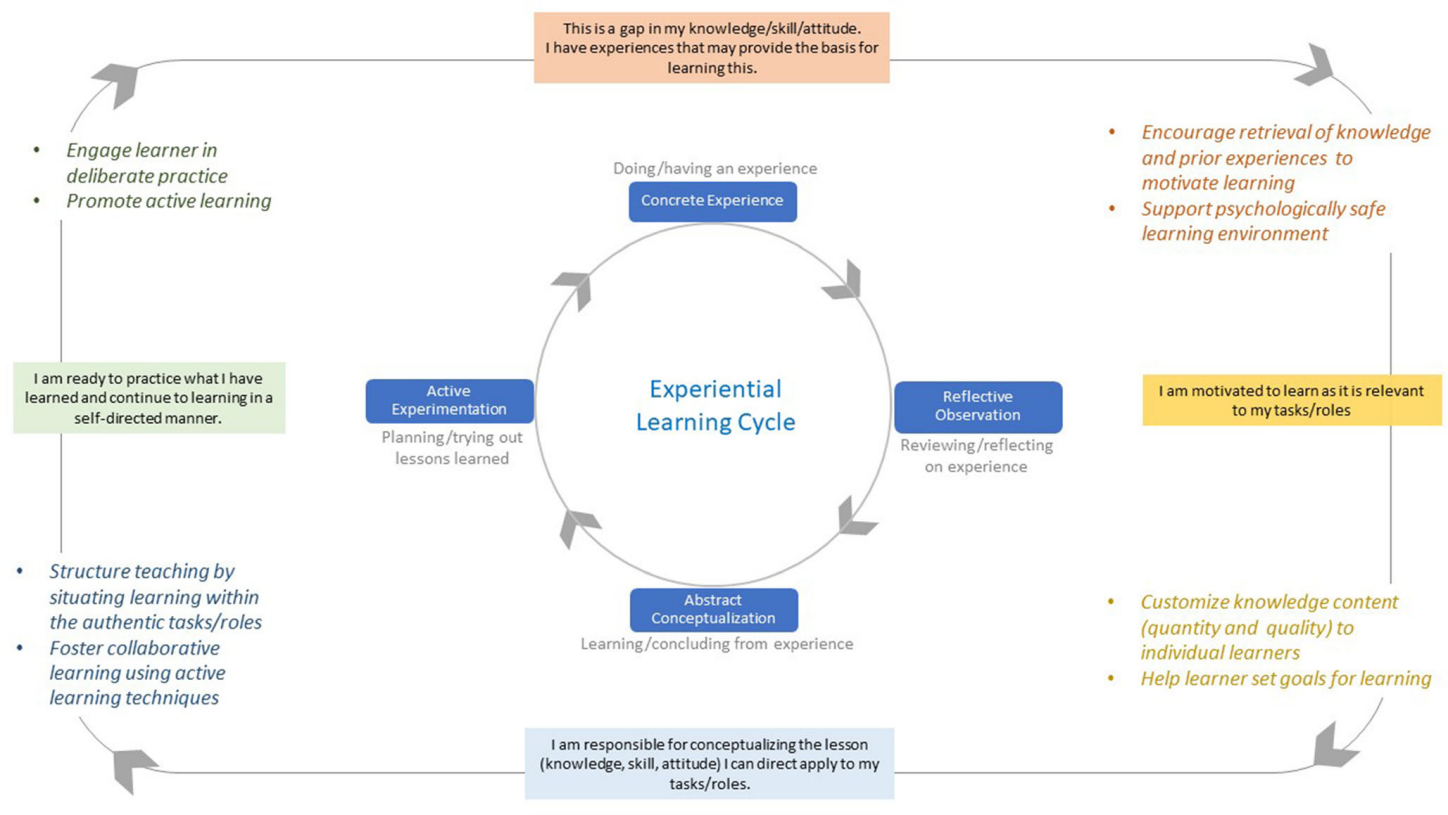
The conceptual model of the “physiology of learning”

The integration of Knowles’ principles with Kolb’s experiential learning model allows for an easy-to-follow guide of effective clinical teaching to support learning in medical students and residents (Fig. 1). We illustrate this with examples how the steps in this model can be executed by busy clinical teachers. This list is by no means exhaustive; it is intended to serve as a starting point for interested readers to explore the suggested literature, and to try out new approaches based on the educational theory provided.

The principles and activities outlined in the conceptual model in Fig. 1 align closely with the kind of things medical learners in the clinical workplace mention with when asked to reflect on good learning experiences [5]. We summarize the ten principles of adult learning clinical teachers can apply to enhance the effectiveness of their teaching in Table 1.

**Table 1:** The principles of adult learning, supporting effective teaching in the clinical workplace

Set one or more clear learning objectives [6, 7] Address learners’ intrinsic motivation to learn by aligning your learning aims to theirs [15] Make sure that the content is relevant to the learners [8] Use learners’ previous experiences to promote reflection and make the topic relevant [15] Pitch the teaching at the right level of previous knowledge [11] Structure your teaching session effectively [11] Show enthusiasm for the topic and for teaching [12] Involve the learners actively [6, 9, 10] Promote reflection by asking questions [14] Provide your learners with repeated constructive feedback [13]	

Kolb’s experiential learning cycle (center) describes the sequence of activities to enable learning for adult learners (outer boxes). In this model, a concrete experience is used to activate the learner’s prior knowledge and to identify a knowledge (or skills, or attitude) gap the learner wishes to fill. Through reflection on the experience, the learner constructs new concepts about the knowledge gap, seeks additional new knowledge from external sources, and determines their application. The learner then applies the learned knowledge in new situations, which provides a new concrete experience to learn from, and starts a new cycle of learning. Different learners may have different preferences at which activity they prefer to enter the learning cycle, but effective learning is supported best by moving through all four activities. Advances in learning sciences offer evidence-based teaching practices (colored texts with adult learning principles) that clinical teachers can employ to enhance their workplace teaching, and to increase the effects of their teaching on their learners.

### Encourage retrieval of knowledge and prior experiences to motivate learning

It is helpful to assess what learners already know as this will help both individual learners and the teacher identify gaps in knowledge, skills, or attitudes. Assessing learners’ current knowledge is particularly useful in the setting with multiple levels of learners, so teachers can customize teaching at a level appropriate for the learners. This can be achieved by using a quiz or self-assessment testing prior to a teaching session, or by Socratic questioning during the teaching encounter. The term “Socratic questioning” does *not* refer to the “pimping” technique in which a supervisor poses a barrage of questions to learners to demean them, but describes an approach to questioning that probes learners’ current understanding to discover contradictions and gaps of understanding, allowing them to express their uncertainties and doubts safely, without the risk of being disparaged, and expand their knowledge [6]. If done properly, teachers can help individual learners activate their prior knowledge or experience to serve as a foundation for the learning session that follows in a homogenous group of learners. Appropriate questioning creates a challenge that prompts learner engagement, particularly if it is sufficiently challenging or difficult to enable the learners to take the next step in their learning.

### Support a psychologically safe learning environment

There is a fine line between effective questioning for inquiry and pimping. A set of rapid-fire questions can easily induce fear and stress among learners. A person’s emotional state plays a significant role in psychological processes of learning [7]. A right amount of stress motivates learning, but too much stress or fear compromises learning. Psychological safety has been shown to be critical to learning [8]. When learners sense interpersonal trust and mutual respect, they feel comfortable to engage in inquiry and discourse freely. Activating existing knowledge and experiences also allows teachers to convey the critical message that learners’ viewpoints and contributions are valued and respected, and teachers can customize the teaching points or learning materials accordingly.

*Example: You could initiate the discourse by questioning interns as a group how the HFNO differs from regular low flow oxygen by nasal canula, allowing each intern to contribute to the answer. Inviting more experienced team members like residents to share their personal experiences or what they have read on the topic can enhance the discourse. The quality of the contributions from these individuals reflects how much they know without putting anyone on the spot.*

### Customize knowledge content to individual learners

To motivate learners, teachers can highlight how the materials are applicable and relevant to learners’ roles and the related tasks. Relevance of knowledge may not always be obvious to novice learners. The assessment from the previous step helps teachers to select teaching points related to the clinical experience encountered. Avoid clinical teaching via a “canned talk”—it will not support clinical experiential learning. Consider the delivery of content in “small chunks” and minimize extraneous loads (e.g., teaching tangential topics or discussing very rare diseases) to reduce cognitive load and optimize learning [9]. Guiding learners to valid and reliable resources for learning assignments allows them to focus on mastering the knowledge rather than wasting cognitive efforts on distinguishing high-quality from low-quality resources. As mentioned earlier, stress or fear can affect learning as negative emotions are also extraneous cognitive load to learning.

### Help learners set goals for learning

Helping learners to set learning goals or providing clear learning objectives for them promotes learners’ motivation and knowledge acquisition and retention [10]. A goal that is too easy or too tedious can be demotivating. To build a motivating learning session, teachers must set a challenging and yet achievable goal, inquire an explicit commitment from learners from the outset, provide intermittent feedback, and adjust or break down the goals (e.g., short-term and long-term goals) according to task complexity [11].

### Foster collaborative learning using active learning techniques

Learning is facilitated when the learner processes the information, not just listens to it [12]. Instead of “spoon feeding” the whole knowledge content, effective teachers engage their audience actively, by asking questions and by providing sufficient support to help learners build on prior knowledge, and move towards new concepts, skills, or understandings. Similar to the scaffolding of a building, teachers can provide a temporary structure that supports their learners in constructing their own new concepts or ideas, by providing basic concepts, content structure, or learning strategies. Scaffolding and active learning techniques help to reduce frustration and intimidation when learners engage in a difficult topic or task, and encourage further self-directed learning.

*Example: You could provide some background knowledge, e.g., on the pathophysiology of bronchiolitis, and then ask an advanced learner to teach basic respiratory physiology (e.g., lung capacity and volumes) or to discuss the most relevant clinical endpoints to be used in HFNO clinical trials, before assigning different learners to explore the topic further (e.g., types of respiratory failure for interns, present results of systematic reviews of clinical trials or of the validity and applicability of evidence in clinical practice guidelines for bronchiolitis for the upper-level resident), and to report back later that day. You may consider guiding learners to high-yield educational resources (e.g., books, online videos) to facilitate efficiency of learning.*

### Structure teaching by situating learning within authentic tasks/roles

Learning is a social activity which should always be situated or attached to the clinical context in which it occurs [13]. In addition, learners will always adapt their learning behavior to what they perceive to be the social norms of the medical profession. Clinical learning environments are perfect to role model collaborative learning wherein each member contributes to and learns from one another to construct meaningful new knowledge. Teachers can foster collaborative learning through active learning strategies (e.g., think-pair-share, debate, problem-solving task) [14, 15]. Such collaborative learning within a clinical department will help to shape the development of the learner’s professional identity as a team member [16]. As the current generation of learners grow up playing games and have gamer’s mindset (e.g., adopt a challenge, prefer options, cultivate connectedness), the use of game elements (e.g., by using a reward system of points, levels, or leaderboards) stimulates engagement and allows learners to explore, fail, and learn within safe boundaries [17].

*Example: To induce active learning, you could divide learners into teams to study and debate about the effective use of HFNO in different situations. If you wish, you can incorporate game elements like using a “challenge of the day” for point collection as individuals or teams. In addition to solving the case at hand, you can bring in other real clinical cases to compare and contrast the large variations in clinical manifestations.*

### Engage learners in deliberate practice

Acquiring competence in complex skills can be promoted through deliberate practice, a process of systematic repeated exposure to the tasks of performing the complex skills, supported by repeated structured constructive feedback [18] Teachers who want their learners to use the knowledge they transferred during a teaching episode will therefore challenge the learners to try it out in practice, reflect on the experience, and ask for feedback.

### Promote active learning

Deliberate practice is, in fact, the repeated progression through Kolb’s learning cycle (Fig. 1). Complex and integrated skills like those involved in communicating with patients [19], or applying evidence-based medicine [20], cannot be learned in a single standalone teaching exercise, but require deliberate practice. Effective clinical teachers therefore promote active learning by encouraging learners to set new learning aims, by keeping an interest in the learner’s application of newly learned knowledge and skills, by providing constructive feedback repeatedly, and by setting a role model of lifelong learning themselves.

*Example: During the 30-min teaching session after handover, you employed some of the strategies outlined in the examples above, to engage the learners actively and to stimulate discussion. You showed enthusiasm, both for the teaching topic and the teaching as an activity. At the end of the 30-min session, you let the learners summarize what they have learned and what they will apply in practice. You provide them with reading materials for further exploration, and you set a date for the follow-up teaching session in which you will discuss what they have read, thought of, and tried out.*

## Data Availability

N/A

## Code availability

N/A
